# Human-Biomonitoring derived exposure and Daily Intakes of Bisphenol A and their associations with neurodevelopmental outcomes among children of the Polish Mother and Child Cohort Study

**DOI:** 10.1186/s12940-021-00777-0

**Published:** 2021-08-25

**Authors:** Mercè Garí, Rebecca Moos, Daniel Bury, Monika Kasper-Sonnenberg, Agnieszka Jankowska, Aleksandra Andysz, Wojciech Hanke, Dennis Nowak, Stephan Bose-O’Reilly, Holger M. Koch, Kinga Polanska

**Affiliations:** 1 Institute and Clinic for Occupational, Social and Environmental Medicine, University Hospital, LMU Munich. Institute of Computational Biology, Helmholtz Zentrum München, Munich, Germany; 2 Institute for Prevention and Occupational Medicine of the German Social Accident Insurance, Institute of the Ruhr-University Bochum (IPA), Bochum, Germany; 3 Department of Environmental and Occupational Health Hazards, Nofer Institute of Occupational Medicine (NIOM), Lodz, Poland; 4 Department of Health and Work Psychology, Nofer Institute of Occupational Medicine (NIOM), Lodz, Poland; 5 Department of Environmental Epidemiology, Nofer Institute of Occupational Medicine (NIOM), Lodz, Poland; 6 Institute and Clinic for Occupational, Social and Environmental Medicine, University Hospital, LMU Munich, Munich, Germany

**Keywords:** Birth cohort, Bisphenol A, Children, Daily Intake, Neurodevelopment, Poland

## Abstract

**Background:**

Bisphenol A (BPA) is an industrial chemical mostly used in the manufacture of plastics, resins and thermal paper. Several studies have reported adverse health effects with BPA exposures, namely metabolic disorders and altered neurodevelopment in children, among others. The aim of this study was to explore BPA exposure, its socio-demographic and life-style related determinants, and its association with neurodevelopmental outcomes in early school age children from Poland.

**Methods:**

A total of 250 urine samples of 7 year-old children from the Polish Mother and Child Cohort Study (REPRO_PL) were analyzed for BPA concentrations using high performance liquid chromatography with online sample clean-up coupled to tandem mass spectrometry (online-SPE-LC-MS/MS). Socio-demographic and lifestyle-related data was collected by questionnaires or additional biomarker measurements. Emotional and behavioral symptoms in children were assessed using mother-reported Strengths and Difficulties Questionnaire (SDQ). Cognitive and psychomotor development was evaluated by Polish adaptation of the Intelligence and Development Scales (IDS) performed by trained psychologists.

**Results:**

Urinary BPA concentrations and back-calculated daily intakes (medians of 1.8 μg/l and 46.3 ng/kg bw/day, respectively) were similar to other European studies. Urinary cotinine levels and body mass index, together with maternal educational level and socio-economic status, were the main determinants of BPA levels in Polish children. After adjusting for confounding factors, BPA has been found to be positively associated with emotional symptoms (β: 0.14, 95% CI: 0.022; 0.27). Cognitive and psychomotor development were not found to be related to BPA levels.

**Conclusions:**

This study represents the first report of BPA levels and their determinants in school age children in Poland. The exposure level was found to be related to child emotional condition, which can have long-term consequences including social functioning and scholastic achievements. Further monitoring of this population in terms of overall chemical exposure is required.

**Supplementary Information:**

The online version contains supplementary material available at 10.1186/s12940-021-00777-0.

## Background

Bisphenol A (BPA) is an organic compound mostly used in the manufacture of plastics, resins and thermal paper since the decade of the 60s [1]. There has been a widespread application of BPA in many daily use products, including food contact materials (*e.g.* reusable bottles, containers for beverages and food), toys, packaging, paints and water pipes, and medical devices [2]. Humans are therefore exposed to BPA through ingestion of contaminated foods and drinks, inhalation and dermal contact. Once in the human body, BPA is mainly metabolized in the liver. BPA glucuronide is the major metabolite along other minor metabolites, including BPA sulfate. The urinary elimination half-life of BPA in humans is estimated to be around 5 h [3].

Bisphenol A has been classified as toxic for human reproduction and identified as an endocrine disrupting chemical (EDC) for human health and the environment [1]. In 2019, the General Court of the EU confirmed that BPA must be listed as a “substance of very high concern” for its hormonal disrupting properties on the human body, upholding a previous decision by the European Chemicals Agency. Consequently, BPA has been already banned or restricted in the EU for some products, such as baby bottles (banned since 2011), toys (migration limit of 0.04 mg/l), plastic bottles and food packaging (banned since 2018 in products for babies and children under the age of three, and for the rest, migration limit of 0.05 mg/kg), and more recently, in thermal paper (since 2020, limit of 0.02%) [4]. In any case, BPA is still allowed to be used in the domain of plastics all over Europe, including Poland.

As in the case of other EDCs, there is particular concern that fetuses, infants or children are more vulnerable to these exposures compared to adults [5]. BPA may interact with a variety of hormonal systems that affect growth, metabolism, and neurodevelopment. The existing studies, including several reviews, have concluded that BPA exposure might be related to neuro-behavioral problems in children [6–11]. However, the results from the existing publications are not fully consistent. The reason lies in methodological differences such as different exposure time (prenatal *vs* infancy *vs* childhood), exposure level, use of variety of tools for measuring neurodevelopmental domains and controlling for confounding variables. Sex-specific directions of the associations cannot be excluded either. The postulated mechanism of BPA effects, even at low doses, is complex and not fully understood. BPA interference with hormonal and genomic regulation is, however, pointed out. BPA can bind not only to nuclear and membrane estrogen receptors but also to thyroid, glucocorticoid and peroxisome proliferator-activated receptors, and it can also interact with steroidogenic enzymes [7, 9–11]. The developing brain is a key target for this compound and thus both prenatal and childhood are sensitive periods of exposure.

The worldwide prevalence of mental disorders in children and adolescents was estimated by Polanczyk et al. in a publication from 2015 [12]. The overall prevalence was reported to be 13.4% (95% CI: 11.3–15.9). Specific mental disorders such as anxiety, depressive disorders or Attention-Deficit/Hyperactivity Disorders (ADHD) had a prevalence of 6.5% (95% CI: 4.7–9.1), 2.6% (95% CI 1.7–3.9) and 3.4% (95% CI 2.6–4.5), respectively [12]. These psychiatric conditions along with altered children’s intellectual potential can impact children’s well-being, scholastic achievement, and social functioning later in life [12].

Children may have higher BPA exposures compared to adults [13]. This can be related to developmentally appropriate differences in diet, behavior, physiology, anatomy, and toxicokinetics. In 2015, within the European framework project DEMOCOPHES, urinary BPA levels were analyzed through a harmonized protocol in mothers and children [14]. Overall, for BPA, the urinary concentrations were similar among both age groups [15]. Although only six countries were selected for the BPA assessment, the results showed few geographical differences [16]. Among the analyzed countries, Slovenia showed the highest BPA levels in children. In addition, this study showed that maximum daily BPA intakes were above the temporary tolerable daily intake (t-TDI) of 4 μg/kg bw/d derived by EFSA [17] in a small subset of children [16]. However, to date there are no or limited available studies on the levels of BPA in Poland, particularly for what concerns children’s exposure and its health consequences. Ongoing European Human Biomonitoring Initiative (project HBM4EU, 2017–2021) give the potential for comparison of exposure levels in European countries (including Poland) based on the standard protocol [18].

Commercial production of BPA in Poland began in 1978, in the Southern village Kędzierzyn-Koźle. The plant was one of the first BPA process technologies in the world [19]. BPA production in Poland is about 12,000 tons/year, although another report estimated a BPA production of 20,000 tons in 2013 [19, 20]. Some Polish scientists have claimed to perform a nation-wide biomonitoring in Poland in order to evaluate the health risks posed by BPA exposure [20]. Other European countries, such as France, Sweden or Germany, have proposed to restrict the use of BPA and investigate more in depth the potential risks of this compound and other similar bisphenols in the environment [4]. The present study is therefore devoted to contribute to fill this gap by the analysis of BPA in urine samples, collected from 250 children from the Polish Mother and Child Cohort (REPRO_PL) study [21]. In addition, we aim to explore the associations of BPA levels and neurodevelopmental outcomes, following previous assessments on this cohort and other environmental exposures such as phthalates [22].

The aims of this study is to characterize the levels and socio-demographic and lifestyle-related determinants of BPA exposure in children from Poland, to calculate their estimated daily intakes, and to explore the associations with neurodevelopmental outcomes including emotional and behavioral symptoms as well as cognitive and psychomotor development.

## Methods

### Study population and sampling

The study is based on the Polish Mother and Child Cohort (REPRO_PL), which is described in detail in previous publications [21, 23, 24]. Briefly, the study comprised three phases covering the prenatal period (phase I: 2007–2011), child examination at the age of 1 and 2 years (phase II: 2008–2013), and child examination at the age of 7 years (phase III: 2014–2019). The REPRO_PL is aimed to assess the impact of a variety of environmental and lifestyle related factors on pregnancy outcomes and children’s health. For this purpose, a sample of the Polish population was selected, sampling pregnant women (and their children) from several regions, including big cities with more than 500 thousand of inhabitants as well as small villages and towns. Out of 407 children who were followed-up until the age of 7 years, spot urine samples from 250 children (61%) collected the day of examination (in the period 2014–15) were randomly selected for the analysis of BPA. Details of that procedure have been published previously [21, 22, 25]. Written informed consent was obtained from the parents of each child before the study, which was approved by the Ethical Committee of the Nofer Institute of Occupational Medicine, Lodz, Poland (Decision No. 22/2014).

### Analysis of Bisphenol A and creatinine

Urine analyses were performed to measure concentrations of BPA as total BPA after enzymatic hydrolyses [26]. The analytical procedure has been described in detail elsewhere [27, 28]. Quality control materials (consisting of four pooled urine samples with different but known concentrations of BPA) were included in each batch of samples. The limit of quantification (LOQ, based on a signal-to-noise ratio of 9) was 0.1 μg/l. The laboratory successfully obtained consecutive certificates for BPA analyses in urine of the external quality assessment Scheme G-Equas (www.g-equas.de) also qualified for BPA analyses within the pan-European HBM Project HBM4EU (www.HBM4EU.eu).

Urinary creatinine was determined with a working range of 0.05–5.00 g creatinine/l by contract analysis in an accredited laboratory at the Nofer Institute of Occupational Medicine (NIOM), Lodz, Poland (Cary 60 UV-Vis spectrometer Agilent Technologies; MS Spektrum, Poland) [29].

### Socio-demographic and lifestyle-related variables

The following socio-demographic information was obtained by questionnaire filled out by mothers at child examination: place of residence (rural, urban; defined using a threshold of 10 thousand of inhabitants); household status (single parenthood, parents living together); number of siblings (none, 1, 2 or more); socio-economic status (SES) of the family (least affluent, affluent and most affluent; defined using a subjective assessment of the mothers with four possible answers: very poor or poor, good and very good, respectively); parental educational level (years of completed education: ≤9, 10–12, > 12); parental occupational activity (yes, no); child sex and age (exact age based on date of examination and date of birth). Parental age was calculated for date at child birth. Data concerning breastfeeding (no: < 2 weeks, short: 2 weeks – 6 months, long: > 6 months) was collected after delivery and at follow-up examinations. Information on children’s passive smoking at the age of 7 years was extracted from cotinine levels in urine analysed by LC-ESI-MS/MS method in an accredited laboratory at NIOM [30, 31] (LOQ was 0.7 μg/l). A cut-off value of 2.1 μg/l for child environmental tobacco smoke exposure (ETS) was selected based on the analysis that was done on the Polish children at similar age as in the current study [31]. Child height and weight were measured at the age of 7 years by trained staff based on standard protocols [21]. Body mass index (BMI) categories (underweight boys < 13.95 kg/m^2^, girls < 13.80 kg/m^2^; recommended weight boys 13.96–18.64 kg/m^2^, girls 13.81–18.19 kg/m^2^; overweight-obesity boys > 18.65 kg/m^2^, girls > 18.20 kg/m^2^) were based on Polish reference data BMI z-scores at the age of 7 years [32]. Considering that in Poland children can start school education when they are 6 or 7 years old (which can potentially impact their exposure level and neurodevelopmental outcomes) that information was included in the models evaluating the association of BPA with neurodevelopmental outcomes. Additional variables considered in the models included mother-reported traumatic events experienced by the child (yes/no), prenatal exposure to tobacco constituents (based on cotinine levels in saliva collected from the mothers during the pregnancy period and measured by LC-ESI-MS/MS method in an accredited laboratory at NIOM, with LOQ 0.4 μg/l [33], and child birth weight and gestational age (based on data from medical records).

### Neurodevelopmental assessments

Child emotional and behavioral symptoms were assessed by the Strengths and Difficulties Questionnaires (SDQ) (www.sdqinfo.com, parent reported) filled in by the mothers at the time as the child examination was performed [34]. The 25 items in the SDQ constitute of five scales (conduct problems, hyperactivity/inattention problems, emotional symptoms, peer relationship problems and prosocial behavior) of five items each. For each of the five scales the score ranges from 0 to 10 if all items are completed. These scores were scaled up pro-rata if at least 3 items were completed. All sub-scale scores excluding prosocial behavior were summed as total difficulties score (range from 0 to 40) to assess the total behavioral problems. Validated, scale specific cut-offs were used to classify children with the symptoms within the borderline or clinical range and within the clinical range only [34]. In addition to the five scales, two amalgamated scales, namely externalizing and internalizing scores, were calculated. Both scores range from 0 to 20. In the case of externalizing scores, conduct and hyperactivity/inattention problems are summed, while for internalizing scores, emotional symptoms and peer relationship problems are combined [35, 36].

Child cognition and psychomotor development was assessed with a Polish adaptation of the Intelligence and Development Scales (IDS) for Children aged 5–10 years, performed by trained and certified psychologists [37]. In the current study, the general intellectual ability (fluid and crystallized intelligence) as well as cognition and mathematical, language and psychomotor skills were evaluated. Reliability for fluid and crystallized intelligence equals 0.94, and for the full scale, 0.96. A more detailed explanation on the neurodevelopmental assessments performed on the REPRO_PL children is found elsewhere [22].

### Daily intake and cumulative risk assessment

Daily intake (DI) of BPA was calculated from urinary levels according to the following equation [16, 38]:

$$\mathrm{DI}=\frac{{\mathrm{BPA}}_{\mathrm{creatinine}}\ast {\mathrm{CE}}_{\mathrm{rate}}}{\mathrm{Fue}\ast \mathrm{bw}}$$

where DI is the daily BPA intake (in μg/kg bw/day); BPA_creatinine_ is the creatinine-adjusted BPA concentration (in μg BPA/g creatinine); CE_rate_ is the child individual body height and gender based reference values for urinary creatinine excretion rate (in g/day) [39]; Fue is the urinary excretion fraction for total BPA (sum of glucuronide, sulfate, and free BPA), assumed to be 1 [27]; and bw is the body weight for each child (in kg).

### Data analysis

Data analysis and graphics were performed using the statistical software R [40] and ggplot package [41]. For descriptive analysis, medians and geometric means (GM) with 95% confidence intervals (CI) of BPA were used. Percentile 95 and maximum values were also reported in the tables. Statistical differences in the covariates between children included or not included in the study were tested for significance using the Chi-square test (Supplementary Material). Two different multivariate linear regression analyses were used: on the one hand, to assess the association of socio-demographic covariates (independent variables) with BPA concentrations (dependent variable); on the other, to assess the relationship between neurodevelopmental outcomes (SDQ and IDS scores, dependent variables) with the BPA levels controlling for socio-demographic and subject characteristics (independent variables). Before inclusion in the models, BPA concentrations were transformed into the natural logarithm and standardized (centred at zero and scaled to two standard deviations) [42].

The first model (aimed to explore the associations of socio-demographic and lifestyle determinants on the levels of BPA) included the following covariates: sex, BMI, place of residence, urinary cotinine levels, SES, maternal educational level and occupational status.

For the second set of models (aimed to assess the relationships between emotional and behavioral symptoms (SDQ) and cognitive and psychomotor development of children (IDS) and BPA levels, controlling for socio-demographic characteristics) multivariate linear regression was used. In addition, other regression models were applied for SDQ: on the one hand, Poisson and negative binomial regression, and on the other, logistic regression for SDQ symptoms dichotomized (both for children within the borderline or clinical range grouped together and compared with children within normal range; and children within clinical range only compared with children within normal range and borderline). The SDQ models were adjusted by children’s sex, age, maternal educational level, socio-economic status, household status, number of siblings, cotinine levels at age 7 and in the 1st trimester of pregnancy, and maternal age, while the IDS model were adjusted by the aforementioned variables (except age at examination, since the test is already standardized for age) in addition to the examiner. All the models underwent sensitivity analysis, including (*e.g.* birth weight and gestational age) or excluding (*e.g.* cotinine levels in pregnancy) certain variables. In addition, we explored the sex differences in the aforementioned associations by adding an interactive term between the sex and the BPA levels. The models were also checked for homoscedasticity/linearity, goodness of fit and independence of predictors using diagnostic plots, the variance inflation factor and the Jarque Bera test, respectively. The final models were selected by both AIC (Akaike Information Criteria) and BIC (Bayesian Information Criteria). Type 1 error was set to 0.05.

## Results

The characteristics of the population are described in Tables 1 and S1. Detailed description of the study population is presented in our previous publications [22, 25]. Except for age at examination (7.2 ± 0.23 years *vs.* 7.5 ± 1.1 years; *p* < 0.05), no differences were found between the subset of children included and not included in the BPA analysis. This analysis included almost similar proportion of boys and girls. Parental’s educational level was high (65% of the mothers and 42% of the fathers declared university degree), mostly represented in the higher SES (78 and 20% of the population indicated affluent and most affluent levels, respectively), and declared high occupational activity (89% of the mothers and 98% of the fathers). Only 10% of the children were not breastfed (or less than 2 weeks), 32% less than 6 months and 58% of the children longer than 6 months. About 40% of the children were exposed to ETS. Six percent of the children were classified as underweight and 17% were overweight or obese.

**Table 1:** Characteristics of the study population (Poland, 2014–15, *n* = 250)

	N (%)
**Sex of the child**	
Female	134 (54)
Male	116 (46)
**Child age at examination**^a^	7.2 ± 0.23
**BMI groups**	
Underweight	15 (6)
Recommended weight	192 (77)
Overweight/Obese	43 (17)
**Place of residence at 7 yr**	
Urban	216 (86)
Rural	34 (14)
**Urinary cotinine levels at 7 yr**	
﻿< 2.1 ng/ml	148 (59)
﻿> 2.1 ng/ml	101 (41)
**Maternal age at delivery**	
< 30 years	155 (62)
> 30 years	95 (38)
**Socioeconomic status of the family at 7 yr**	
Most affluent (Very good)	50 (20)
Affluent (Good)	195 (78)
Least affluent (Poor or very poor)	5 (2)
**Maternal educational level at 7 yr** **(years of completed education)**	
≤ 9	5 (2)
10–12	83 (33)
> 12	162 (65)
**Maternal occupational status at 7 yr**	
No	28 (11)
Yes	219 (89)

^a^ Mean ± SD

Neurodevelopmental outcomes, including emotional and behavioral symptoms (SDQ) and cognitive and psychomotor development (IDS) of children, are presented in Table 2 and described in detail in a precedent publication [22]. Briefly, about 25% of the children were classified within borderline/clinical range of conduct problems, emotional symptoms and hyperactivity/inattention scales (Table 2). Peer relationship problems were noted among 18% of the studied population whereas for a 7% of the children the scores for prosocial behavior scales were within borderline and clinical range. Intellectual efficiency and psychomotor skills were within the normal range.

**Table 2:** Child neurodevelopmental scores for behavioral problems (SDQ) and cognitive and psychomotor development (IDS) in children from Poland (n = 250)

Neurodevelopmental outcomes	Mean (±SD)	Range	Borderline/Clinical (%)
**Behavioral scales (SDQ)**			
Prosocial behavior	8 (±2)	4–10	7
Conduct problems	2 (±1)	0–7	28
Emotional Symptoms	2 (±2)	0–8	25
Hyperactivity/Inattention problems	4 (±3)	0–10	24
Peer relationship problems	1 (±2)	0–7	18
Total difficulties	9 (±5)	0–26	19
Internalizing scores	4 (±3)	0–13	NA
Externalizing scores	6 (±3)	0–15	NA
**Intellectual ability (IDS)**			
Fluid intelligence (IQ)	104 (±14)	56–136	NA
Crystallized intelligence (IQ)	104 (±15)	60–142	NA
Cognition	73 (±12)	17–99	NA
Mathematical skills	11 (±3)	3–19	NA
Motor skills	30 (±6)	11–43	NA
Language skills	21 (±5)	6–32	NA

Urinary BPA was determined in all of the studied 7-year old children from the Polish REPRO_PL cohort (*n* = 250), with BPA below the LOQ in only one sample. The concentrations ranged between <LOQ (treated as LOQ/2, *i.e.* 0.05 μg/l) and 53.1 μg/l, with a median of 1.8 μg/l (GM of 1.9 μg/l) and a 95th percentile of 10.7 μg/l (Table 3). Creatinine-adjusted concentrations ranged between 0.081 μg/g creatinine and 92.5 μg/g creatinine, with a median level of 2.6 μg/g creatinine (Table 3). Table 4 shows an international comparison of urinary BPA levels reported from different studies.

**Table 3:** BPA urinary concentrations (in μg/l and μg/g creatinine) and daily intakes (in ng/kg bw/day) in children from Poland (REPRO_PL birth cohort, n = 250)

	GM^**a**^ (95% CI^**b**^)	Median	P25^**c**^	P75^**d**^	P95^**e**^	Range
Urinary BPA concentrations (*μg/l)*	1.9 (1.7–2.1)	1.8	1.1	2.8	10.7	0.05–53.1
Urinary BPA concentrations (*μg/g creatinine)*	2.8 (2.5–3.1)	2.6	1.7	4.0	16.3	0.081–92.5
Daily Intakes of BPA (*ng/kg bw/day)*	50.0 (44.5–56.1)	46.3	28.8	75.9	301.7	1.6–1840

^a^ GM: Geometric Mean; ^b^ CI: Confidence Interval; ^c^ P25: Percentile 25th; ^d^ P75: Percentile 75th; ^e^ P95: Percentile 95th

**Table 4:** Comparison of BPA concentrations in different children populations worldwide

Location	Year	n	Age	BPA (***μ***g/l)^**a**^	Reference
Poland	2014–15	250	7	1.9 [1.7–2.1]	This study
Europe	2011–12	653	5–12	2.0 [1.8–2.2]	[16]
Germany	2006–2010	465	8–10	2.2 [2.0–2.4]	[43]
Portugal	2014–15	110	4–18	1.6 [1.2–2.1]	[44]
Denmark	2011	143	6–11	1.7 [1.1–3.7]^b^	[45]
Norway	2012	56	6–12	3.1 [1.8–4.1]^b^	[46]
Slovenia	2011–12	145	6–11	2.4 [0.67–4.6]^b^	[47]
Turkey	2015–16	125	3–6	0.60^b^	[48]
US	2013–14	409	6–11	1.4 [1.3–1.6]	[49]
Ohio, US	2003–14	222	8	1.6 [1.0–3.6]^b^	[50]
Canada	2013–16	77	3–4	1.0 [0.6–1.5]	[51]
Brazil	2012–13	300	6–14	1.7 [0.30–35.9]^c^	[52]
China	2009–10	412	7	2.7 [2.2–3.2]	[53]
Hong Kong	2016	31	4–6	1.7 [0.72–2.3]^b^	[54]
South Korea	2016	162	7–9	0.60 [0.34–1.2]^b^	[55]
India	2012–13	76	2–14	5.1 [0.070–41.4]^c^	[56]

^a^ GM [95% CI] ^b^ Median [25th–75th percentiles] ^c^ GM [Range]

**Fig. 1:**
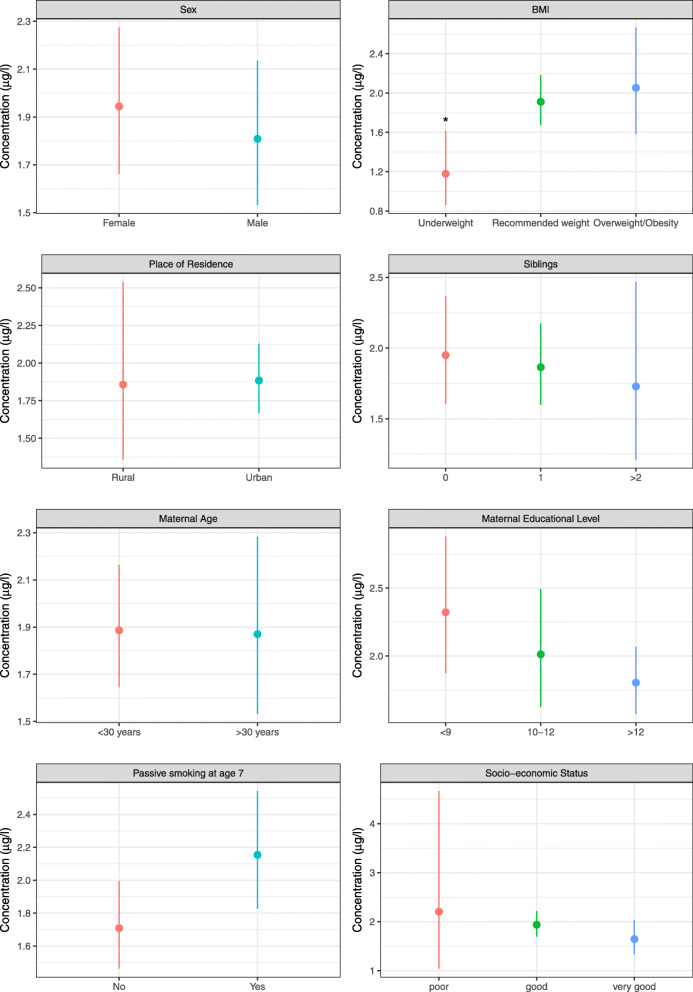
Geometric means and 95% confidence intervals (μg/l) of BPA concentrations in 7-year old children for several socio-demographic characteristics: children’s sex and BMI, number of siblings, place of residence, passive smoking at age 7, maternal age and educational level, and socio-economic status

**Fig. 2:**
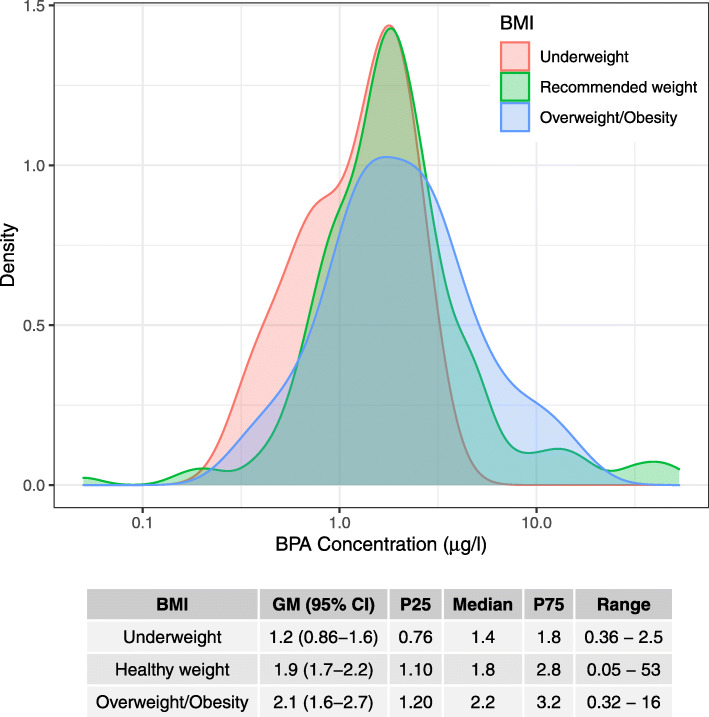
Density plot of BPA levels for each z-score body mass index category: underweight, healthy weight, and overweight/obesity

Figure 1 shows the univariate associations of several socio-demographic and lifestyle-related determinants on the levels of BPA in studied children. Specifically, BPA concentrations were higher among children exposed to ETS (Fig. 1). Maternal educational level and SES were inversely associated to BPA concentrations in children, with highest BPA levels among children whose mothers had low educational levels or those belonging to the least affluent socio-economic status (Fig. 1). The concentrations of BPA were significantly lower (*p*-value < 0.05, Fig. 1) in children with underweight. Figure 2 shows a more detailed distribution of the BPA levels by z-score BMI groups. Specifically, BPA concentrations are widespread within children in the healthy weight group. Children classified as overweighted and obese usually ranged in the highest BPA levels, while BPA in underweighted children was within the low range (Fig. 2). Creatinine-adjusted BPA concentrations for each BMI group are found in Table S2 (Supporting Information). Other sociodemographic determinants, such as children’s sex, number of siblings, place of residence or maternal age did not show any significant difference with the levels of BPA (Fig. 1). Multivariate regression models confirmed some of the aforementioned trends (Fig. S1). Specifically, children whose urinary cotinine levels were higher than 2.1 ng/ml showed higher BPA levels, and also underweight was inversely related to BPA concentrations, being both variables statistically significant at a 10% confidence level (p-value < 0.1; Fig. S1).

**Fig. 3:**
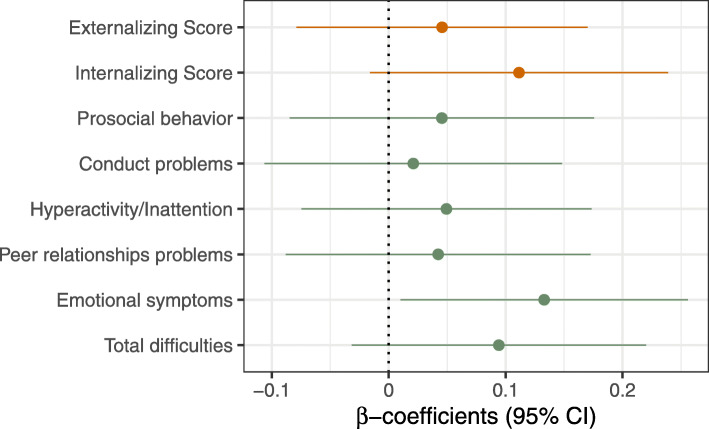
Standardized beta-coefficients from multivariate regression models for BPA concentrations on the behavioral scales (SDQ) in children at 7 years of age. Models are adjusted for child’s sex and age at examination, household status, SES, maternal educational level, maternal age at birth, number of siblings, cotinine levels during 1st trimester of pregnancy and in children at 7 years of age

**Fig. 4:**
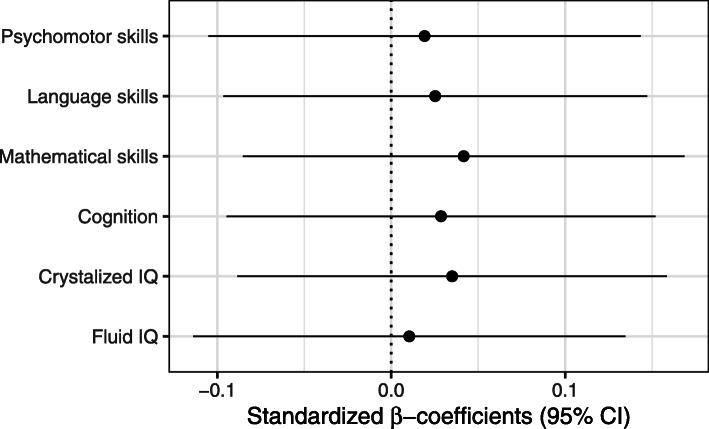
Standardized beta-coefficients from multivariate regression models for BPA concentrations on intelligence and development scales (IDS) in children at 7 years of age. Models are adjusted for child’s sex, examiner, household status, SES, maternal educational level, maternal age at birth, number of siblings, cotinine levels during 1st trimester of pregnancy and in children at 7 years of age

Figures 3 and 4 show the results for the association of BPA exposure with children’s a) emotional and behavioral outcomes and b) intelligence, cognition, mathematical, language and psychomotor skills considered as continuous variables (SDQ and IDS, respectively). After controlling for sociodemographic and lifestyle-related factors, BPA was found to be positively associated with emotional symptoms (β: 0.14, 95% CI: 0.021; 0.27). The other scales, namely conduct problems, hyperactivity/inattention problems and peer relationships problems, together with the prosocial behavior, as well as the total difficulties score, were not found to be significantly associated with the BPA concentrations in 7-year old children (Fig. 3). When the symptoms were dichotomized (within borderline/clinical range vs. normal range or clinical range vs. normal/borderline range) the observed effect estimates on the emotional symptoms were not maintained (Fig. S4). Cognitive and psychomotor development measured with IDS were not found to be associated with BPA levels after controlling for covariates. The results did not change in the sensitivity analyses with inclusion or exclusion of certain variables (Figs. S5 and S7 for SDQ and IDS, respectively). On the other hand, different effects depending on the children’s sex were not found. This was assessed using an interactive term between sex and BPA levels on the associations with neurodevelopmental outcomes (data not shown).

## Discussion

In general, the BPA concentrations in Poland (1.9 μg/l) ranged within similar levels as those found in other European school-age children (Table 4). For 653 European children (DEMOCOPHES study) a median level of 2.0 μg/l was reported, with some differences among European member states [16]. For instance, Slovenia and Belgium were the two countries with the highest BPA levels (2.6 μg/l and 2.4 μg/l, respectively), in comparison to the other European locations analyzed in the report, such as Denmark, Spain, Luxembourg and Sweden (ranging from 1.5 μg/l to 1.9 μg/l) [16]. Other European studies performed in Portugal (1.6 μg/l), Denmark (1.7 μg/l), Slovenia (2.4 μg/l) and in a German cohort (2.2 μg/l) reported similar BPA levels [43–45, 47]. Only one study performed in 56 children aged 6–12 years from Norway reported higher BPA concentrations (3.1 μg/l) [46]. However, the differences regarding BPA concentrations found between those studies cannot be attributed to different population-exposure levels but to the variability in sample size, children’s age, analytical procedure and sampling period. The concentrations in Poland are also in the same range than those found in US studies, including the 2013–14 NHANES report and the Ohio cohort (US), as well as in Hong Kong and Brazil, with GM concentrations ranging from 1.4 μg/l to 1.7 μg/l [49, 52, 54, 57], but twice higher than those found in Canada, Turkey or South Korea (0.6–1.0 μg/l) [48, 51, 55]. The concentrations were, however, much higher in studies performed in Asian populations, such as one conducted in China (2.7 μg/l) and another in India (5.1 μg/l) [53, 56].

BPA has been gradually banned in many countries since 2009, especially in food-related products for children, which has resulted in an effective decrease of BPA exposures in child populations [58, 59]. BPA exposures in the US also declined during the time period from 2003 to 2012 [60]. Therefore different time points of sample collection, together with other relevant factors mentioned above, might contribute to the small differences in concentrations found in the reported countries and continents (Table 4) [61]. The overall trend indicates a decreasing BPA exposures among the general population.

For our study population we calculated oral daily BPA intakes using the urinary excretion fraction (Fue) for BPA. The Fue was set to a value of 1 based on human metabolism studies indicating that almost 100% of the orally administered BPA is excreted via urine within 24 h after exposure [3]. In our study, the median daily BPA intake was 46.3 ng/kg bw/day, ranging from 1.6 ng/kg bw/day and 1840 ng/kg bw/day (Table 3). None of the studied children had BPA intakes above the European t-TDI established by EFSA in 2015, set to 4 μg/kg bw/day [17]. Thus, the median daily intake resembled roughly 1%, and the highest calculated daily intake resembled roughly 50% of this EFSA t-TDI with even bigger margins for the US EPA oral reference dose of 50 μg/kg bw/day. In the worldwide context, the global BPA exposure level was estimated to be 60.1 ng/kg bw/day [62], while in Europe, children’s median BPA DIs ranged from 31.3 ng/kg bw/day to 38.2 ng/kg bw/day [16, 44, 45]. In the DEMOCOPHES study, however, the report showed DIs estimations above the t-TDI of 4 μg/kg bw/day in a subset of children from Denmark and Belgium [16].

Regarding educational level and socio-economic status, the European DEMOCOPHES study also found higher BPA levels among children from families with primary education in relation to secondary or tertiary education, similarly to the present results [16]. In the HOME cohort study performed in Ohio (US), the authors also found that household annual incomes and maternal education were associated with BPA levels in children, with the same trends [57]. Conversely, a recent study performed on six European cohorts found lower BPA levels in low education groups [63]. To our knowledge, none of the reports on BPA in children has found an association with sex, although a recent study performed in Turkey found higher concentrations among girls in relation to boys [16, 48]. In relation to the body mass index, a study performed in Portuguese children found an inversed pattern, in which children with obesity had lower BPA levels than underweight/healthy weight children [44]. However, the children from this study were enrolled in a dietary program for weight loss/management, and therefore, apart from physical characteristics (*e.g.* BMI), diet or other determinants might influence the BPA exposure patterns in children. In fact, a recent report based on six NHANES examination cycles between 2003 and 2014 was not able to find any significant association between BPA in urine and serum lipids in children and do not hint to metabolic effects by BPA exposure [64].

In our study, BPA was found to be positively associated with emotional symptoms. What is worth emphasizing is that this result was independent of previous traumatic experiences in a child’s life that is more likely expected to affect the emotional health of the children (data not shown). Even though the observed effect of BPA exposure on children emotional development is rather small, still it can have the long-term consequences looking at their social functioning and scholastic achievements. The result of the review from 2017 [8] pointed out that there is a relationship between BPA exposure both during prenatal and childhood period and negative behavioral outcomes including emotional development. However the existing studies are not fully conclusive. A study by Braun et al. (HOME cohort) assessed the effect of childhood BPA exposure on behavior and executive functions in children aged 3 years and found that the associations were largely null and not modified by child gender [65]. Harley et al. (CHAMACOS cohort) observed a relationship between BPA exposure at age 5 years and increased externalizing behaviors, including conduct problems, in girls at age 7 years and increased internalizing behaviors and inattention and hyperactivity behaviors in boys and girls at age 7 years [66]. Roen et al., when studying the CCCEH cohort (follow-up), showed that BPA levels in the urine of children measured at 3 and 5 years were related to the behavior of girls aged 7–9 years - higher levels of anxiety, depression and aggression were observed in them [67]. In a cross-sectional study, Hong et al. showed that BPA levels in the urine of girls and boys aged 8–10 years were associated with higher levels of anxiety and depression [68]. In the Perera et al. publication (based on CCCEH cohort), childhood exposure was not significantly related to the measured anxiety and depression symptoms in children aged 10–12 years [69]. Perez-Lobato, when studying boys aged 9–11 from the INMA cohort, showed increased problems with the internalization of behavior [70]. Stacy et al. reported sex-dependent associations between BPA and child neurobehavior [50]. A 10-fold increase in 8-year BPA was associated with more externalizing behaviors in boys, but not in girls [50]. Two recent studies based on data from several mother-child cohorts in Europe have also evaluated the associations between simultaneous exposure to a wide range of environmental chemicals (including BPA exposure) and child behavior [71, 72]. BPA urinary concentrations during the prenatal period were associated with higher (worse) scores on the externalizing behavior sub-scale evaluated between 3 and 7 years of age [71] although no associations were observed for the postnatal period [72]. Discrepancies in the literature could be partly due to differences in the characteristics of the study populations (*e.g.* socio-economic status) or study designs, including differences in the timing of exposure or outcome assessment, neurobehavioral assessments tools, BPA exposure misclassification, or irrelevant time frames of exposure assessment. Nevertheless, as pointed out in the existing research, BPA exposure is more frequently associated with externalizing/internalizing problems than cognitive outcomes. Both internalizing and externalizing symptoms seem to coexist and a consistent pattern in term of sexual differences is not present [11].

The future analyses should use the potential of prospective mother-child cohort study design and look more deeply at long-term consequences of BPA exposure. Thus, the continued follow-up of existing cohorts should be a priority for future studies in this area. Moreover, considering the real exposure scenario, the studies should jointly consider both vulnerable periods of exposure (prenatal and postnatal) as well as simultaneous exposures to different chemical classes. Taking into account that BPA has been classified as an endocrine-disrupting chemical, the differential effect of sex and BPA levels on neurodevelopmental outcomes should be explorer more in depth. Finally, the future research should put more efforts to focus on understanding the mechanisms that may explain the observed patterns of association, such as DNA methylation, immune dysfunction, systemic inflammation, oxidative stress, and endocrine and metabolic disruption [10]. All those above-mentioned require large sample thus an exposome-based approach considering joint analyses based on data from existing birth cohorts should be strengthen in future research.

The current study is based on a well-defined child cohort from Poland. The follow-up was performed accurately. Child mental health was assessed based on widely used standardized and validated questionnaire that distinguish children with and without emotional and behavioral symptoms. Moreover, quantitatively assessed data allows examination of the symptoms in the whole spectrum, which do often not qualify for clinical diagnosis but still might have a great impact on an individual’s behavioral health and can result in long-term consequences (*e.g.*, school achievements). In addition, child cognitive and psychomotor assessments were performed by trained and certified psychologists. IDS has a high reliability as well as high correlation with analogous WISC-R scales [73].

The laboratory analysis was performed with the highest level of internal and external quality assurance, ensuring with utmost accuracy and comparability of urinary BPA concentrations [26, 74]. We have also excluded external contamination by analyzing the conjugation status of BPA in the urine samples. Above 90% of total BPA was present in its conjugated form (data not shown). Still, we have to acknowledge that BPA is a non-persistent chemical, rapidly eliminated via urine. Exposure assessments based on a single spot of urine may thus wrongly classify the children’s individual overall or long term BPA exposure. Although sex-specificity of the effects of BPA and child neurodevelopment has been underlined in existing studies, we were not able to find such association, probably due to the available sample size. On the statistical side, although the models were validated and underwent sensitivity analysis, since they were adjusted for numerous variables, the possibility of residual or unmeasured confounding cannot be excluded. In addition, other chemical exposures such as phthalates (which were measured in a previous study [25]) were not considered in the current analysis. Finally, causality cannot be inferred due to the observational nature of the current study and needs to be confirmed through the application of other methods to strengthen causal inference. Due to the cross-sectional design of this analysis, single exposure measures of BPA along with the low exposure levels are limited to capture effects of BPA on the health status during childhood. In addition, there is no data on previous children’s exposure to BPA. Long-term exposure measures for BPA, starting with prenatal exposure estimations and repeated measures in critical developmental time frames during childhood are highly warranted to solve the problems with the short-term exposure estimations for BPA.

## Conclusions

This study provides current biomonitoring data of Bisphenol A in urine of 250 children from Poland. The concentrations and daily intakes of BPA among this Polish cohort were in concordance with other European studies. Exposure with environmental tobacco smoke and body weight were associated with BPA in 7-year old children. Specifically, higher urinary cotinine concentrations were associated with higher BPA levels, and underweight children showed lower BPA levels than healthy weight or overweight/obese children. Furhermore, this study was able to find a positive association between BPA concentrations in 7-year old children and emotional symptoms. Some neurodevelopmental problems in children might be associated to the exposure of nonpersistent chemicals such as BPA. More efforts on understanding the mechanisms that may explain the observed associations should be explored, specifically those related to gene-environment interactions.

## Supplementary Information

**Additional file 1.**

## Data Availability

The datasets used and/or analysed during the current study are available from the corresponding author on reasonable request.

## Abbreviations

ADHD: Attention-Deficit/Hyperactivity Disorders
AIC: Akaike Information Criteria
BIC: Bayesian Information Criteria
BMI: Body Mass Index
BPA: Bisphenol A
bw: Body weight
CE: Creatinine excretion
CI: Confidence Interval
DI: Daily Intake
ECHA: European Chemical Association
EDC: Endocrine disrupting chemical
EFSA: European Food Safety Authority
ETS: Environmental Tobacco Smoke
Fue: Urinary excretion fraction
GM: Geometric mean
HBM: Human Biomonitoring
IDS: Intelligence and Development Scales
LC-ESI-MS/MS: Liquid chromatography electrospray ionization tandem mass spectrometry
LOQ: Limit of quantification
MS: Mass Spectrometry
OR: Odds Ratio
REPRO_PL: Polish Mother and Child Cohort Study
SES: Socio-economic status
SDQ: Strengths and Difficulties Questionnaire
SPE-LC-MS/MS: Solid phase extraction coupled to liquid chromatography and tandem mass spectrometry
t-TDI: Temporary tolerable Daily Intake
USEPA: United States Environmental Protection Agency
WISC-R: Wechsler Intelligence Scales for Children-Revised
