# Correction: Human-Biomonitoring derived exposure and Daily Intakes of Bisphenol A and their associations with neurodevelopmental outcomes among children of the Polish Mother and Child Cohort Study

**DOI:** 10.1186/s12940-023-00977-w

**Published:** 2023-03-13

**Authors:** Mercè Garí, Rebecca Moos, Daniel Bury, Monika Kasper-Sonnenberg, Agnieszka Jankowska, Aleksandra Andysz, Wojciech Hanke, Dennis Nowak, Stephan Bose-O’Reilly, Holger M. Koch, Kinga Polańska

**Affiliations:** 1 Institute and Clinic for Occupational, Social and Environmental Medicine, University Hospital, LMU Munich. Institute of Computational Biology, Helmholtz Zentrum München, Munich, Germany; 2 Institute for Prevention and Occupational Medicine of the German Social Accident Insurance, Institute of the Ruhr-University Bochum (IPA), Bochum, Germany; 3 Department of Environmental and Occupational Health Hazards, Nofer Institute of Occupational Medicine (NIOM), Lodz, Poland; 4 Department of Health and Work Psychology, Nofer Institute of Occupational Medicine (NIOM), Lodz, Poland; 5 Department of Environmental Epidemiology, Nofer Institute of Occupational Medicine (NIOM), Lodz, Poland; 6 Institute and Clinic for Occupational, Social and Environmental Medicine, University Hospital, LMU Munich, Munich, Germany

**Correction: Environ Health 20, 95 (2021)**

**https://doi.org/10.1186/s12940-021-00777-0**

Following publication of the original article [1], the authors reported an error in Table 3. The mistake refers specifically to the units in the daily intakes of bisphenol A in children from Poland: instead of ‘ug/kg bw/day’, it should be ‘ng/kg bw/day’. The mistake only appears twice in Table 3: in the table title and in the last row of the table. For the rest of the article (text, other tables and figures), the units are correct.

The original article has been updated to correct this.
