# Supplementary material for: Human-Biomonitoring derived exposure and Daily Intakes of Bisphenol A and their associations with neurodevelopmental outcomes among children of the Polish Mother and Child Cohort Study
Source: Environ Health. 2021 Aug 25;20:95. doi: 10.1186/s12940-021-00777-0 (PMC8390261; doi:10.1186/s12940-021-00777-0)

Supporting Information

Human-Biomonitoring derived Exposure and Daily Intakes of Bisphenol A and their Associations with Neurodevelopmental Outcomes among Children of the Polish Mother and Child Cohort Study

Mercè Garí, Rebecca Moos, Daniel Bury, Monika Kasper-Sonnenberg, Agnieszka Jankowska, Aleksandra Andysz, Wojciech Hanke, Dennis Nowak, Stephan Bose-O'Reilly, Holger M. Koch, Kinga Polanska

Content:

Supporting Information Tables

Table S1: Characteristics of the study population…………………………………………….….. 2
Table S2: BPA urinary concentrations in creatinine-adjusted………………………………..… 4
Table S3: Multivariate Linear Regression Models applied on SDQ…………………………..….. 5
Table S4: Multivariate Linear Regression Models applied on IDS…………………………..… 6
Table S5: Additional Multivariate Regression Models applied on SDQ……………………… 7

Supporting Information Figures

Figure S1: Beta-coefficients form Multivariate Regression models on BPA ….………………. 8
Figure S2: Descriptive analysis on SDQ neurodevelopmental outcomes ……..………………. 9
Figure S3: Descriptive analysis on IDS neurodevelopmental outcomes …...………………….. 10
Figure S4: Multivariate Regression Models applied on SDQ ..……………………………….... 11
Figure S5: Sensitivity Analysis for SDQ ….……………………………………………………. 12
Figure S6: Sensitivity Analysis for IDS ..………………………………………………………. 13

Table S1. Characteristics of the study population according to participation in the BPA analysis (Poland, 2014-15).

	**Children included**[a] **n=250**	**Children not included n=157**	**p-value**[b]
	N (%)	N (%)	
**Sex of the child**			0.4
Female	134 (54)	77 (49)	
Male	116 (46)	80 (51)	
**Child age at examination (yr)**[c]	7.2±0.23	7.5±1.1	<0.05
**BMI groups**			0.2
Underweight	15 (6)	16 (10)	
Recommended weight	192 (77)	118 (75)	
Overweight/Obese	43 (17)	23 (15)	
**Place of residence at 7 yr**			0.4
Urban	216 (86)	130 (83)	
Rural	34 (14)	27 (17)	
**Urinary cotinine levels at 7 yr**			0.4
**<2.1 ng/ml**	148 (59)	118 (75)	
**≥2.1 ng/ml**	101 (41)	39 (25)	
**Maternal age at delivery**			0.9
<30 yr	155 (62)	96 (61)	
>30 yr	95 (38)	61 (39)	
**Parity at child's birth**			0.8
None	138 (55)	83 (56)	
One	80 (32)	44 (29)	
Two or more	32 (13)	22 (15)	
**Number of siblings at 7 yr**			0.4
None	78 (31)	51 (32)	
One	149 (60)	85 (54)	
Two or more	22 (9)	21 (13)	
**Breastfeeding**[d]			NA
No (<2 weeks)	20 (10)	NA	
Short (2 weeks – 6 months)	62 (32)	NA	
Long (>6 months)	113 (58)	NA	
**Socioeconomic status of the family at 7 yr**			0.7
Most affluent (Very good)	50 (20)	38 (24)	
Affluent (Good)	195 (78)	116 (74)	
Least affluent (Poor or very poor)	5 (2)	3 (2)	
**Maternal educational level at 7yr (years of completed education)**			0.3
≤ 9 yr	5 (2)	8 (5)	
10-12 yr	83 (33)	34 (22)	
>12 yr	162 (65)	115 (73)	

**Maternal occupational status at 7yr**			1
No	28 (11)	18 (11)	
Yes	219 (89)	139 (89)	
**Paternal educational level at 7 yr (years of completed education)**			0.09
≤ 9 yr	10 (4)	6 (5)	
10-12 yr	135 (54)	50 (42)	
>12 yr	103 (42)	63 (53)	
**Paternal occupational status at 7yr**			NA
No	5 (2)	2 (2)	
Yes	227 (98)	117 (98)	
**Household status**			0.4
Parents living together	213 (86)	141 (90)	
Single parent household	34 (14)	16 (10)	
**Gestational Age (weeks)**[c]	39.2 (±1.5)	39.3 (±1.4)	0.2
**Body weight (g)**[c]	3384 (±479)	3430 (±450)	0.3
**Cotinine levels in pregnancy**			0.7
**<10 ng/ml**	224 (90)	138 (88)	
**≥10 ng/ml**	26 (10)	19 (12)	
**Age at school attendance**			0.6
6 years old	128 (52)	76 (48)	
7 years old	119 (48)	81 (52)	
**Traumatic events**			1
No	218 (88)	137 (87)	
Yes	30 (12)	20 (13)	

[a] BPA concentrations available. [b] *p*-value from Chi-square t-test for comparison of children included and not included in the study. [c] Mean ± SD. [d] Most of the children not included in the study at 7 years of age were not evaluated at age 1 or 2, hence no answer on the breastfeeding status or duration was asked. Therefore, for this variable, data was not available, and shown as NA.

Table S2. BPA urinary concentrations (in µg/g creatinine) in children from Poland, for the total cohort and BMI groups (REPRO_PL birth cohort, n=250).

**BPA**	**GM[a] (95% CI[b])**	P25[c]	Median	P75[d]	Range
Total population	2.8 (2.5 – 3.1)	1.7	2.6	4.0	0.05 – 93
Underweight children	2.5 (1.7 – 3.5)	1.4	2.3	3.6	0.85 – 9.0
Recommended weight children	2.8 (2.4 – 3.2)	1.7	2.6	4.0	0.05 – 93
Overweight/Obese children	2.8 (2.1 – 3.7)	1.6	2.5	4.4	0.38 – 26

[a] GM: Geometric Mean; [b] CI: Confidence Interval; [c] P25: Percentile 25th; [d] P95: Percentile 75th.

Table S3. Multivariate Linear Regression Models applied on SDQ.

	**Main model**[a]	**S.A. 1**[b]	**S.A. 2**[c]
	**β-coeff (95% CI)**[d]	**β-coeff (95% CI)**[d]	**β-coeff (95% CI)**[d]
Internalizing score	0.11 (-0.016;0.24)	0.12 (-0.00065;0.25)	0.11 (-0.022;0.24)
Externalizing score	0.046 (-0.079;0.17)	0.054 (-0.067;0.18)	0.045 (-0.080;0.17)
Prosocial behavior	0.045 (-0.085;0.18)	0.05 (-0.076;0.18)	0.045 (-0.087;0.18)
Conduct problems	0.021 (-0.11;0.15)	0.022 (-0.10;0.15)	0.024 (-0.10;0.15)
Hyperactivity/Inattention	0.049 (-0.075;0.17)	0.061 (-0.060;0.18)	0.047 (-0.079;0.17)
Peer relationship problems	0.042 (-0.088;0.17)	0.037 (-0.090;0.16)	0.038 (-0.094;0.17)
Emotional symptoms	**0.13 (0.010;0.26)**[e]	**0.15 (0.035;0.27)**[e]	**0.13 (0.0048;0.25)**[e]
Total difficulties	0.094 (-0.032;0.22)	0.11 (-0.016;0.23)	0.091 (-0.036;0.22)

[a] Main Model: The models have been adjusted by children's age and sex, maternal age and educational level, socio-economic status, household status, number of siblings, cotinine levels at 1st trimester of pregnancy and in children's at 7 years of age. [b] S.A 1.: Sensitivity Analysis 1. The models have been adjusted by the same variables as in the main model (first column) except cotinine levels at 1st trimester of pregnancy. [c] S.A 2.: Sensitivity Analysis 2. The models have been adjusted by the same variables as in the main model (first column), in addition to birth weight and gestational age. [d] β-coefficient and 95% Confidence Intervals. [e] In bold, statistically significant results (p-value <0.05).

Table S4. Multivariate Linear Regression Models applied on IDS.

	**Main model**[a]	**S.A. 1**[b]	**S.A. 2**[c]
	**β-coeff (95% CI)**[d]	**β-coeff (95% CI)**[d]	**β-coeff (95% CI)**[d]
Cognition	0.029 (-0.095;0.15)	0.025 (-0.096;0.15)	0.028 (-0.097;0.15)
Fluid IQ	0.010 (-0.11;0.13)	0.012 (-0.11;0.13)	0.0087 (-0.12;0.13)
Crystallized IQ	0.035 (-0.088;0.16)	0.038 (-0.083;0.16)	0.034 (-0.091;0.16)
Mathematical skills	0.042 (-0.085;0.17)	0.032 (-0.092;0.16)	0.047 (-0.082;0.18)
Language skills	0.025 (-0.096;0.15)	0.034 (-0.085;0.15)	0.021 (-0.10;0.14)
Psychomotor skills	0.019 (-0.11;0.14)	0.025 (-0.095;0.15)	0.035 (-0.091;0.16)

[a] Main Model: The models have been adjusted by children's sex, maternal age and educational level, socio-economic status, household status, number of siblings, cotinine levels at 1st trimester of pregnancy and in children's at 7 years of age. [b] S.A 1.: Sensitivity Analysis 1. The models have been adjusted by the same variables as in the main model, except for cotinine levels at 1st trimester of pregnancy. [c] S.A 2.: Sensitivity Analysis 2. The models have been adjusted by the same variables as in the main model, in addition to birth weight and gestational age. [d] Standardized β-coefficients and 95% Confidence Intervals (CI).

Table S5. Additional Multivariate Regression Models applied on SDQ, including Poisson, Negative Binomial (NB) and Logistic Regression.

	**Poisson Model**[a]	**NB Model**[b]	**Logistic Model (normal *vs.* borderline/clinical)**[c]	**Logistic Model (normal/borderline *vs.* clinical)**[d]
	OR (95% CI)[e]	OR (95% CI)[e]	OR (95% CI)[e]	OR (95% CI)[e]
Internalizing score	**1.2 (1.0;1.4)**[f]	1.2 (0.99;1.5)	–	–
Externalizing score	1.1 (0.95;1.2)	1.1 (0.91;1.2)	–	–
Prosocial behavior	1.0 (0.93;1.1)	1.0 (0.93;1.1)	0.72 (0.29;1.8)	0.24 (0.023;2.6)
Conduct problems	1.0 (0.85;1.2)	1.0 (0.85;1.3)	0.98 (0.55;1.8)	0.94 (0.39;2.2)
Hyperactivity/Inattention	1.1 (0.94;1.2)	1.1 (0.91;1.3)	1.2 (0.67;2.3)	1.0 (0.48;2.1)
Peer relationship problems	1.1 (0.89;1.4)	1.1 (0.83;1.5)	1.2 (0.64;2.3)	1.7 (0.73;3.7)
Emotional symptoms	**1.3 (1.1;1.5)**[f]	**1.3 (1.0;1.6)**[f]	1.4 (0.78;2.6)	1.6 (0.77;3.3)
Total difficulties	**1.1 (1.0;1.2)**[f]	1.1 (0.97;1.3)	1.2 (0.61;2.3)	1.3 (0.57;3)

[a] Results of the Multivariate Linear Regression using Poisson distribution. [b] Results of the Multivariate Linear Regression using negative binomial (NB) distribution. [c] Results of the Multivariate Logistic Regression on the normal vs. borderline+clinical outcomes. [d] Results of the Multivariate Logistic Regression Model on the normal+borderline vs. clinical outcomes. All the models were adjusted by children's age and sex, socio-economic status, maternal age, maternal educational level, household status, number of siblings and cotinine levels at pregnancy during the 1st trimester and in children at age 7. [e] Odds Ratio (OR) and 95% Confidence Intervals (CI). [f] In bold, statistically significant results (p-value <0.05).

Figure S1. Beta-coefficients from multivariate regression models for several socio-demographic and individual characteristics. Models were adjusted by sex, body mass index, place of residence, urinary cotinine levels, socio-economic status, maternal occupational status and educational level. All the variables were kept as categorical variables, so the reference individual would be a male child with healthy weight, living in a urban area, whose urinary cotinine level is <2.1 ng/ml, classified within the lowest SES, and whose mother is not working and has the lowest educational level.

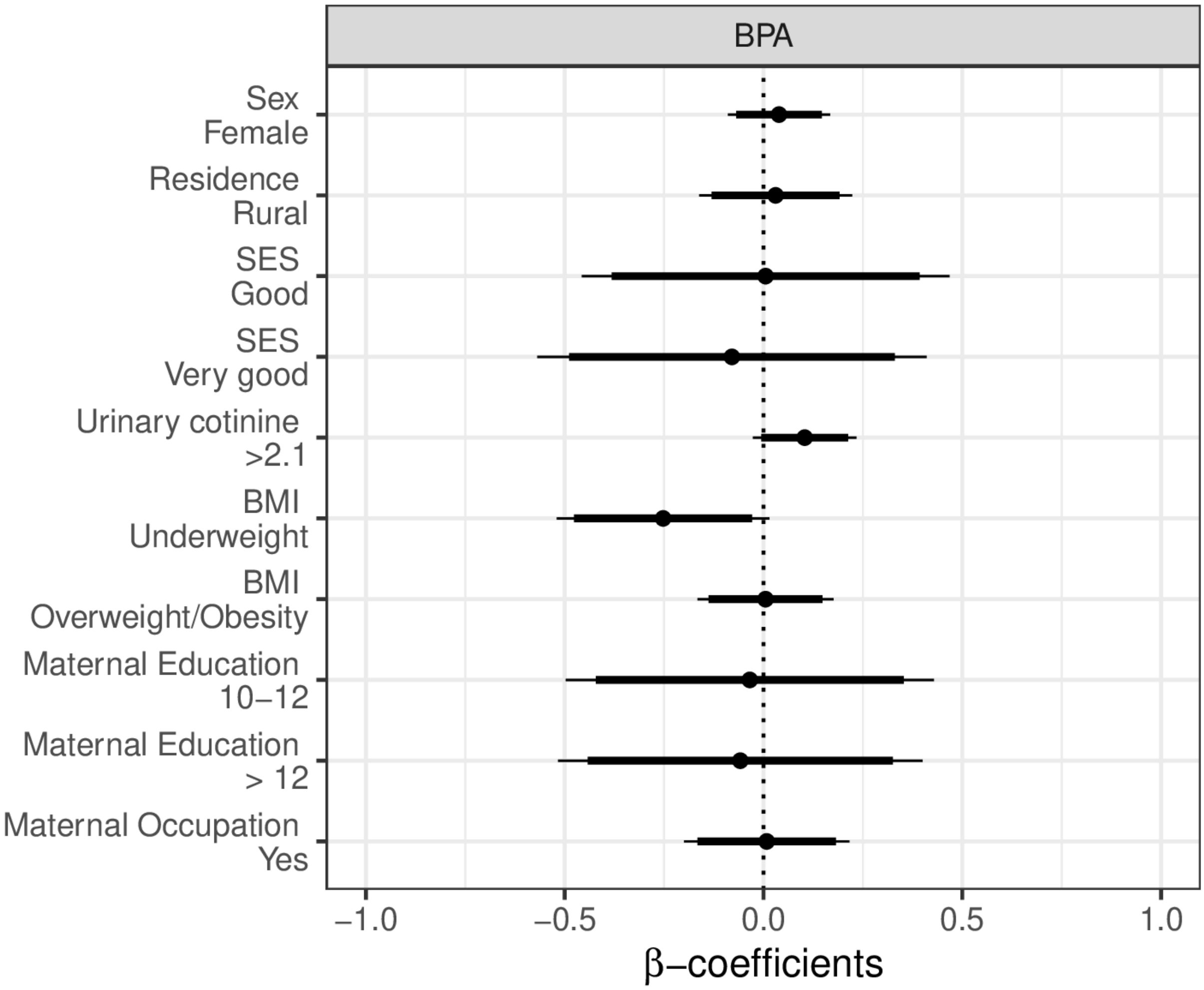

Figure S2. Descriptive analysis of SDQ neurodevelopmental outcomes in the Polish children (n=250).

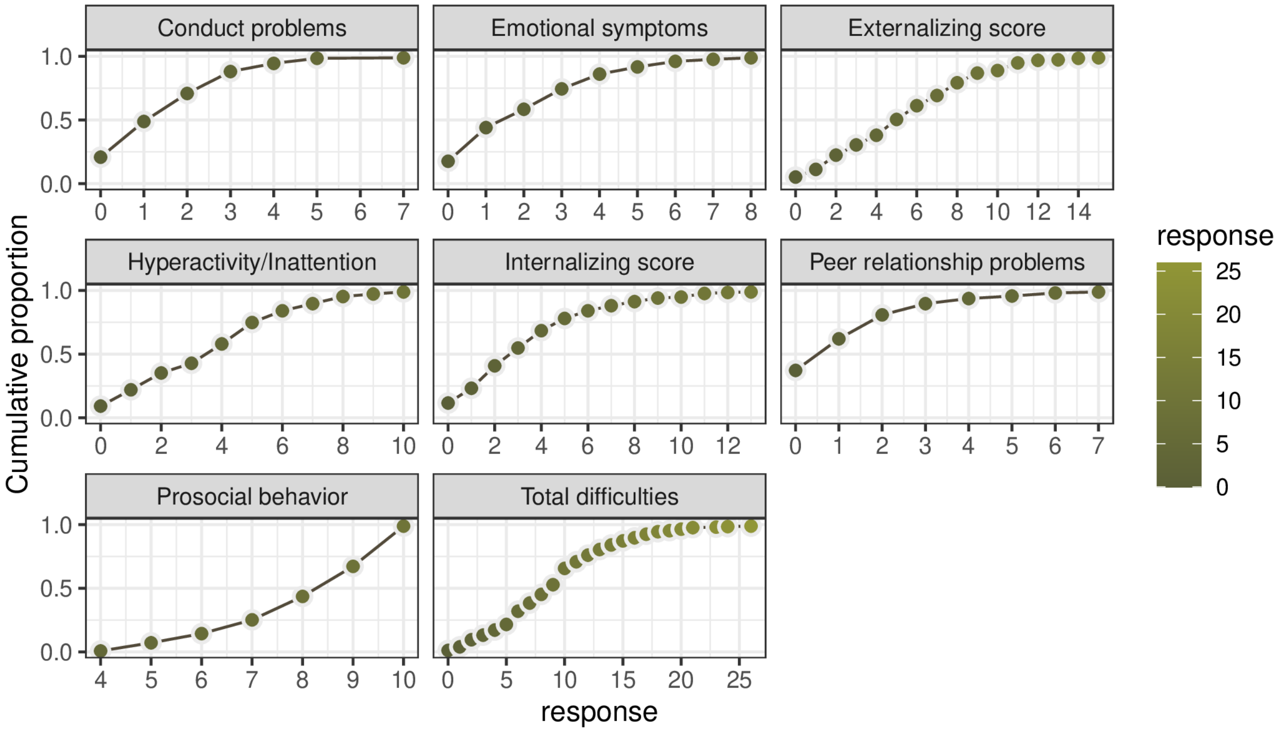

Figure S3. Descriptive analysis of IDS neurodevelopmental outcomes in the Polish children (n=250).

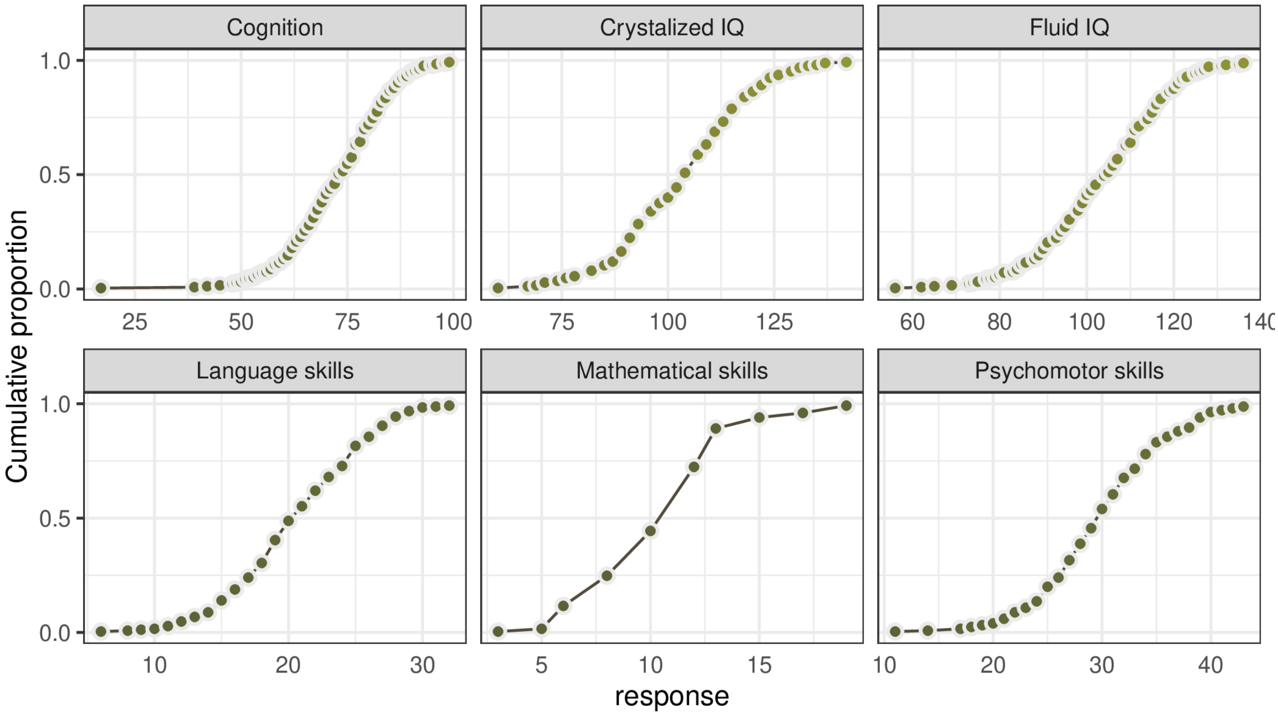

Figure S4. Odds Ratios (OR) and 95% confidence intervals (CI) from different multivariate regression models for BPA concentrations on the behavioral problems scales (SDQ) in children at 7 years of age. A) Multivariate linear regression model using the Poisson distribution. B) Multivariate linear regression model using the negative binomial distribution. C) Multivariate logistic regression on the normal vs. borderline+clinical outcomes. D) Multivariate logistic regression on the normal+borderline vs. clinical outcomes. In all the previous cases, the models were adjusted by the same variables used in the main model, as follows: children's age and sex, socio-economic status, maternal age, maternal educational level, household status, number of siblings and cotinine levels during 1st trimester of pregnancy and in children at age 7.

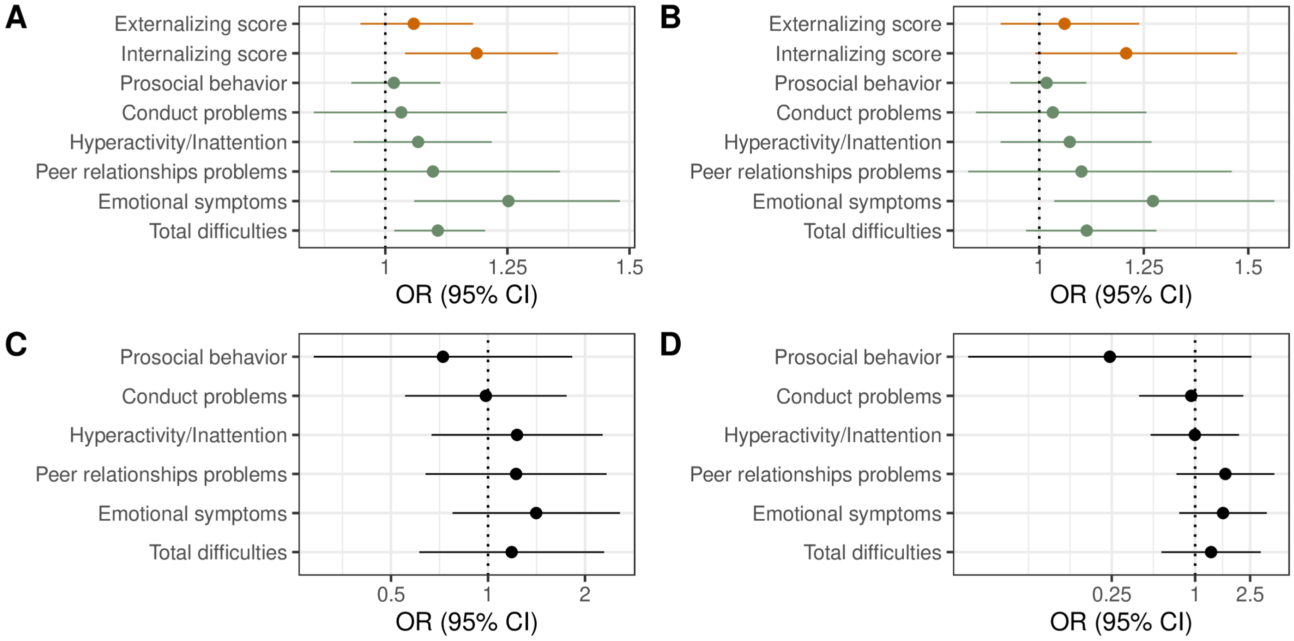

Figure S5. Sensitivity analysis for SDQ. A) The models were adjusted by children's age and sex, socio-economic status, maternal age, maternal educational level, household status, number of siblings and cotinine levels in children at age 7. B) Models were adjusted by children's age and sex, socio-economic status, maternal age, maternal educational level, household status, number of siblings, cotinine levels during 1st trimester of pregnancy and in children at age 7, and birth weight and gestational age.

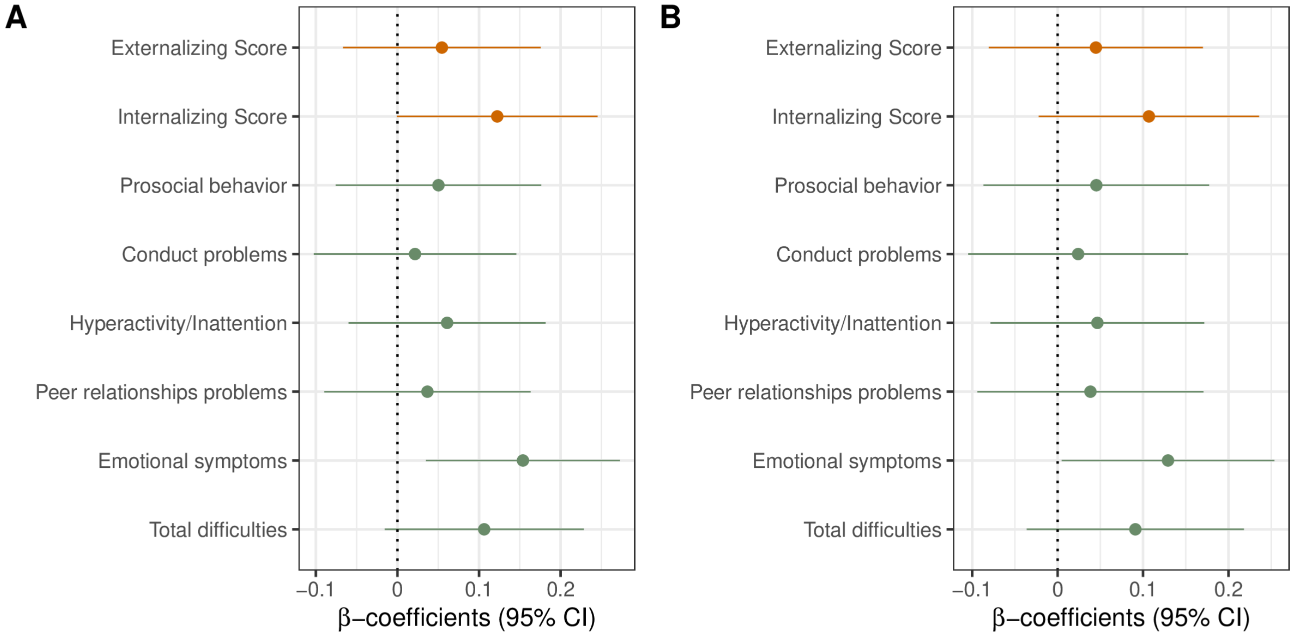

Figure S6. Sensitivity analysis for IDS. A) The models were adjusted by children's sex, socio-economic status, maternal age, maternal educational level, household status, number of siblings and cotinine levels in children at age 7. B) The models were adjusted by children's sex, socio-economic status, maternal age, maternal educational level, household status, number of siblings, cotinine levels during 1st trimester of pregnancy and in children at age 7, and birth weight and gestational age.

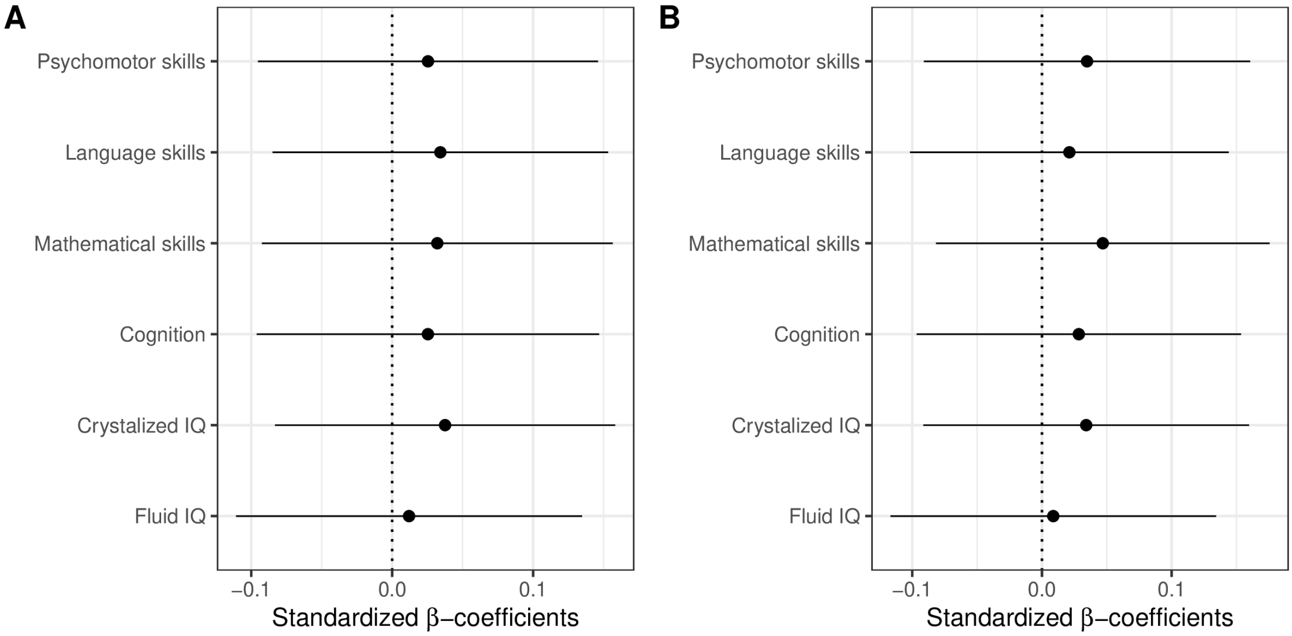

Supplement: Supplementary file 1 — Additional file 1. [file 12940_2021_777_MOESM1_ESM.pdf]
